# Land slide disaster in eastern Uganda: rapid assessment of water, sanitation and hygiene situation in Bulucheke camp, Bududa district

**DOI:** 10.1186/1476-069X-10-38

**Published:** 2011-05-14

**Authors:** Lynn M Atuyambe, Michael Ediau, Christopher G Orach, Monica Musenero, William Bazeyo

**Affiliations:** 1 Makerere University School of Public Health, Department of Community Health and Behavioural Sciences, P.O. Box 7072, Kampala, Uganda; 2 Makerere University School of Public Health/CDC Fellowship Program, Kampala, Uganda; 3 Ministry of Health Uganda, Integrated Disease Surveillance, Kampala, Uganda; 4 Makerere University School of Public Health, Department of Disease Control and Environmental Health, Kampala, Uganda

## Abstract

**Background:**

On 1^st ^March 2010, a major landslide occurred on Mt. Elgon in Eastern Uganda. This was triggered by heavy rains that lasted over three months. The landslide buried three villages in Bududa district, killing over 400 and displacing an estimate of 5,000 people. A comprehensive assessment of water, sanitation and hygiene was urgently needed to inform interventions by the Ministries of Health, and Relief, Disaster Preparedness and Refugees, Uganda.

**Methods:**

This was a cross-sectional study where both qualitative and quantitative data were collected two weeks after the disaster. Quantitative interviews involved 397 heads of households and qualitative methods comprised of 27 Key Informant interviews, four focus group discussions and observations. The survey quantified water safety (collection, treatment, storage) and hygiene practices. This was supplemented and triangulated with qualitative data that focused on community perceptions and beliefs regarding water and sanitation needs and practices. Quantitative data was entered in Epi-Info Version 3.2.2 software and then exported to SPSS Version 12 for analysis. Summary statistics and proportions were generated and bi-variable analysis performed for selected variables. Associations were assessed using odds ratios at 95% confidence intervals. Qualitative data was analyzed using content analysis.

**Results:**

Qualitative results showed that there were strong traditional beliefs governing water use and human excreta disposal. The use of river Manafwa water for household consumption was observed to potentially lead to disease outbreaks. Water from this river was reported tastier and the community culturally saw no need to boil drinking water. Latrines were few (23 for 5000 people), shallow, dirty (70% reported flies, 60% fecal littering), not separated by sex and had limited privacy and no light at night. This affected their use. Males were 3 times more likely to wash hands with soap after latrine use than females (OR = 3.584, 95%CI: 1.658-7.748). Of the 90% respondents who indicated that they always washed hands after latrine use, 76% said they used water and soap. Observations showed that water and soap were inconsistently available at the hand washing facilities. This situation influenced people's sanitation and hygiene behaviours. Nearly half (48%) indicated that at least a member of their household had fallen sick at least once since arrival at the camp.

**Conclusion:**

There was inadequate access to safe water in the camp. Pit-latrines were inadequate, poorly maintained and not user-friendly for most people. Responsible authorities should design means of increasing and sustaining access to safe water, increase sanitation facilities and continuously educate the public on the need to observe good hygiene practices.

## Background

On Monday 1^st ^March, 2010, the Mt. Elgon region of Bududa District in Eastern Uganda experienced a major landslide of unprecedented magnitude in the history Uganda. The landslide, which was triggered by heavy rains lasting over three months, buried three villages (Kubehwo, Namakansa, Nametsi) in Bumayoka sub-county, killed over about 400 people [1] and left 5000 others displaced. The displaced population was temporarily relocated to a camp in Bulucheke sub-county head quarters, 7 km from the site of the disaster. This kind of destruction both to human lives, property and the environment had never occurred on Mt. Elgon before.

Human vulnerability to any disaster is a complex phenomenon with social, economic, health, and cultural dimensions [2]. Specifically, landslides usually cause displacement and often are accompanied by hydro-meteorological events such as floods. Population activities such as the cutting of trees without replacement, agricultural and human settlement facilitate landslides occurrence. Displacement disrupts public health systems, and may lead to inadequate safe water and poor sanitation. This often leads to high morbidity and mortality in the camps [3] due to communicable diseases such as diarrhea.

Communicable diseases have been documented to cause the highest morbidity in refugee, migrant and displaced populations, mainly due to diarrheal diseases such as cholera and dysentery; acute respiratory infection, measles, malaria, with HIV/AIDS and tuberculosis becoming increasingly important [4,5]. The excess morbidity and mortality caused by communicable diseases during emergencies is largely avoidable, when appropriate interventions are put in place. Experience has shown that, when these interventions are implemented in a timely and coordinated manner, deaths and disease are substantially reduced [6]. The risk factors that promote communicable diseases include mass population movement and resettlement in temporary locations, overcrowding, economic and environmental degradation, scarcity of safe water, poor sanitation and waste management, absence of shelter, and poor access to health care [6]. In such circumstances, the public health infrastructure is overwhelmed and prevention services are hampered.

Diarrhoeal diseases are a major cause of morbidity and mortality in emergencies. These diseases mainly result from inadequate quality and quantity of water, substandard and insufficient sanitation facilities, overcrowding, poor hygiene, and scarcity of soap. In camp situations, diarrhoeal diseases account for more than 40% of these deaths in the acute phase of an emergency, with over 80% of these deaths occurring in children aged less than two years. Good planning where public health measures such as appropriate camp site location, availability of clean water, good sanitation and personal hygiene, and health education are critical in controlling spread of diarrheal diseases [6].

Immediately following the Bududa disaster and establishment of the Buluchecke Internally Displaced Persons (IDP) camp, a number of agencies, such as The Uganda Red Cross Society, UNICEF, Ministry of Health, Bududa district health department, Oxfam, Foundation for Development of Needy Communities, World Health Organization, Ministry of Water and Environment, Indian Association of Uganda and Uganda People's Defense Forces moved in to provide emergency interventions in areas of water, sanitation, hygiene and health services promotion. The interventions included provision of safe water, sanitation facilities, hygiene education and promotion. Provision of care and treatment services to the sick was done. A number of the organizations also facilitated the process by providing drugs and other medical supplies and facilities like ambulances. Uganda Red Cross Society acted as the lead agency in providing these emergency relief services.

Soon after the disaster, there was an initial rapid multi-agency situation analysis conducted. This was coordinated by the office of the Prime Minister, Uganda. Our team had access to the report of that rapid situational analysis while designing this survey. However, in the following days the situation evolved rapidly with the number of displaced people growing and putting pressure on the available services. It was therefore evident that a comprehensive assessment of the water, sanitation and hygiene practices was urgently needed to inform the interventions. This data was required to inform government ministries (Ministry of Health and the Ministry of Relief, Disaster Preparedness and Refugees) as well as inform other development partners on the appropriateness of current and future interventions. Makerere University School of Public Health (MakSPH) in collaboration with Ministry of Health (MoH) therefore sanctioned this study to assess the availability and access to safe water and sanitation facilities, as well as hygiene needs and practices among the encamped population.

## Methods

### Design, area, setting and study population

**Figure 1:**
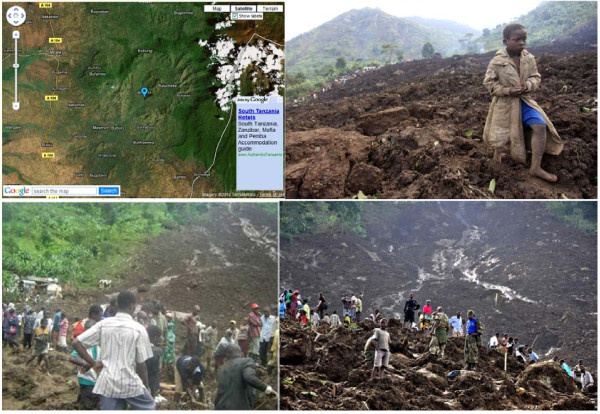
**Map and photographs of Bududa and the landslide scene epicenter, Eastern Uganda**.

This was a cross-sectional study where both qualitative and quantitative data was collected 13 days after the disaster struck. Bududa District located in the eastern region of Uganda is a relatively new district carved out of Manafwa district in 2006. Bududa district is bordered by Manafwa district to the south, Mbale district to the west, Sironko district to the north and by the Republic of Kenya to the east. The coordinates of the district are: 01 01N, 34 20E, and it lie at an average altitude of 1,800 meters (5,900 ft) above sea level (Figure 1). The district headquarters at Bududa are located approximately 23 kilometres by road southeast of Mbale, the largest town in the sub-region. The district comprises of one county, seven sub-counties and one town council. It has unique relief consisting of ridges, cliffs and bamboo forest. In 2006, the district population was estimated at about 146,000, with a male to female ratio of 1:1. The most common language used by inhabitants is Lumasaba. The study population included adults in the camp, relief staff, district and local leaders, and health workers.

### Data collection methods

The qualitative methods comprised Focus Group Discussions (FGDs), Key Informant (KI) interviews and observations. An example of some of the key questions asked in the FGDs and KIs are presented (Appendix 1).

### Focus group discussions

Four focus groups (2 female, 2 male) each with 7-11 participants, were held in the camp. The participants were recruited with the help of the camp and tent leaders. Research assistants (moderator, recorder) with a Bachelors degree in Environmental Health Sciences who were familiar with the local cultural context and spoke the local language were recruited to facilitate the FGDs. These graduate assistants were re-trained in FGD technique and oriented on the FGD guide. Discussions focused on water use, sanitation and hygiene practices in the camp. All FGDs were tape recorded (with consent) and transcribed into English thereafter. Focus group discussions took about one hour thirty minutes each on average. During data collection phase debrief meetings were held at the end of each day to ensure good quality data. Unexpected emerging issues were discussed and followed up in subsequent FGDs.

### Key Informant Interviews

Semi-structured interviews were held with 27 key informants (KIs) in the districts. Key informants included health care providers, NGO and other humanitarian agency workers, district health officials, camp, community leaders and political leaders. They were selected purposefully. First, using our local knowledge of the subject and geographical area, we identified some that we thought would be relevant. Then, at the end of each interview we used the snowball technique to identify the next respondent. We asked interviewees to recommend to us two to three possible respondents and their contacts. We then updated our list of respondents and contacted to make appointment for the interviews. Interviews focused on broad issues and implications for adequacy of water supply and use, sanitation and hygiene practices. These interviews were conducted by experienced social scientists with background training in Public Health in complex emergency situations.

### Observations

Systematic observations were made in the camp using a checklist. They focused on water access, hygiene and sanitation practices as well as sanitation infrastructure. We constructed an observation checklist and performed the observations. We identified key areas to observe such as the water sources (tank water and River Manafwa), latrines, and washing facilities after latrine use and refuse disposal. In addition, we also observed some scenarios such as 'infants and children defecating' and the related practices especially caretakers' handling and disposal of fecal waste. Observations were systematic in that they were only made during mornings and late afternoon when there was high use of sanitation and water facilities for a period of five days.

### Quantitative survey

For the survey, we applied probability proportional to size sampling technique. Our calculated sample size was 384. However, we anticipated non-response of 5% therefore our ultimate sample size was 403. We finally achieved a sample of 397. All the tents in the camp were considered in the assessment. A list of all inhabitants and the families (households) therein in the 53 tents was obtained from the camp commandants. These tents were both large and small in size with households ranging from 5 to 16. The biggest tent had about 70 occupants. Using this list, a sampling interval of households was determined and respondents (households' heads) were identified and interviewed using pre-designed semi-structured questionnaire. Therefore, tents that had a higher number of households had a higher probability of households being selected. Interviewer administered questionnaires that lasted about 30 minutes each on average, covered parameters related to safe water, sanitation and hygiene practices. The study explored availability and access to safe water and sanitation facilities. The camp hygiene practices and needs were also investigated. Thematic areas addressed quantitatively were: water, sanitation and hygiene. Under water storage, we had structured question like: What does the family use to store drinking water in? Is this water covered? Is the water container clean? Is the water treated? Regarding excreta disposal, some questions in the questionnaire were: Would you say that you or a member of your family has access to a latrine? Do members of the family use the latrine? Have you observed the following in/around the latrine? a) Flies b) Littering of faeces c) Smell; Do you wash your hand after visiting a latrine? Where do the children in this tent defecate? Do you clean your child's bottom after defecation? If yes, what do you use? We also had a questions on outbreak detection like 'Are you aware of any one (adult or child) who has been passing watery stools for about 3 or more times a day?' A concluding question was 'In your opinion would recommend improving the living situation in the camp?' was included at the end of the questionnaire.

### Data management and analysis

Qualitative data was analyzed using manifest content analysis technique. This type of analysis refers to a process where analysis of the appearance of a particular word or content in textual material is done. Description of the visible, obvious components of text and what the text says in the transcripts is taken into account [7,8]. We read through all the transcripts several times while making notes and jotting in the transcript. All data were logged into a matrix and frequency of responses made. Issues that commonly appeared were therefore closely examined and tracked in the transcripts as stipulated by Graneheim and Lundman, 2004 [8]. Observation data were triangulated with KI and FGD data. This process gave deep insight of the situation and was useful in validating and interpreting the results. Thematic areas that emerged are presented in this article. Quantitative data was entered and cleaned using Epi-Info Version 3.2.2 software and then exported to SPSS Version 12 for analysis. Summary statistics and proportions were generated and bi-variable analysis performed for selected variables. Associations were assessed using odds ratios at 95% confidence intervals.

### Quality control measures

Five research assistants (RAs) were recruited and trained for one day on how to collect quality quantitative data. They were fluent in the local language (Lumasaba) and English. The questionnaires were pre-tested for one day and edited where necessary to cover identified gaps. Meetings were also held with research assistants on daily basis to counter any challenges that were met during data collection process. The study instruments were reviewed by the steering committee that had expertise in health and emergencies from the MakSPH, MoH and Ministry of Relief, Disaster, Preparedness and Relief. During data collection, supervision was done continuously. The team that collected qualitative data was also oriented on the tools used. This team comprised people experienced in conducting qualitative interviews. Research assistants conducted KI interviews in pairs. This helped to ensure that one RA had ample time to engage the KI while the other took notes. All KIs were audio taped and transcribed thereafter. This ensured that the entire interview was captured.

### Ethical considerations

After the disaster, a technical committee was constituted to respond to this emergency. It included staff from the Ministry of Health (epidemiology unit), Office of the Prime Minister -Uganda, the Ministry of Relief, Disaster Preparedness and Refugees, and the Makerere University School of Public Health. Bearing in mind the urgency of the data and the operational nature of this work, it was decided that there was no need to subject the study to the Institutional Review Committee. However, this technical team reviewed the protocol and all the tools, gave comments and approved them. Besides, the purpose and objectives of the assessment were clearly explained to the Bududa district and Bulucheke Sub-country Administration, as well as the Technical health staff who included the District Health Officer (DHO). Permission to conduct the assessment was granted. Before each interview, the purpose of the study was explained to each respondent, and verbal consent obtained. Participants were informed that there were minimal or no risk to their participation in the study, that participation was voluntary, confidential and that they could withdraw their participation anytime during the interview. During interviews we ensured that the respondent was only with the researcher and Focus group participants were informed not to reveal participants' views outside the discussion forum. Data collected was delinked from the transcripts and questionnaire.

## Results

### Demographic characteristics

The key socio-demographic parameters of respondents are summarized in table 1. Majority of the respondents were females (79%). The mean age of respondents was 35 (Min 18, max 80) with most respondents aged between 20 and 40 years (65%). The majority of respondents, (70.5%) attained only primary education with 20% having no formal education at all. Only one (0.3%) respondent had attained tertiary education. On average, a household had 6 people (min 1, max 19).

**Table 1:** Socio-demographic characteristics

Variables	Frequency (n = 397)	Percentage (%)
Sex of respondents		

Males	85	21.4

Females	312	78.6

Age groups		

Less or equal to 20	44	11.1

21-30	132	33.2

31-40	124	31.2

41-50	54	13.6

51-60	28	7.1

≥61	12	3.0

Education level		

No formal education	77	19.4

Primary	280	70.5

Secondary	39	9.8

Tertiary	1	0.3

Number of people in the household		

1-5	190	47.9

6-10	184	46.3

≥11	23	5.8

### Availability and Access to water

The main source of safe water was tank water. Tank water was supplied from National Water and Sewerage Corporation (NWSC) in Mbale town, and transported to the camp using two water tankers. This water was then put into two storage tanks of 10,000 litres each and placed about 60 meters away from the camp. The other sources of water were river and unprotected springs. From key informant interviews and observations, it was evident that that people were also using water from the nearby river Manafwa, reported to be very accessible and always available for various purposes including drinking, washing, bathing, swimming and cooking. A lot of human activities such as bathing, washing clothes, children playing in the water were observed at the river. These activities potentially could cause water contamination. We also established that initially, bottled water was distributed to the people in the camp for drinking, however, by the time of this assessment this had stopped. Gravity water scheme which was expected to supply safe and reliable water was not yet operational by the time of study although construction was underway. This scheme would capture water from the slopes of Mt. Elgon, get stored in a reservoir and then supply to the lower land communities including Bulucheke where the camp was situated.

From the KIs and FGDs, it was established that water was equally accessed by all people in the camp, with each person having access to at least twenty litres of water per day. Water was mostly collected by female adults, because the available collecting containers were twenty-litre containers that were too heavy for young children to carry. In addition, some KIs mentioned that the community believed that it was the women's duty to fetch water.

Regarding distance to water collection points data obtained from FGDs, KI and exploratory walks concur that the estimated distance to the two water sources (water tank and river Manafwa) was 100 meters. Focus groups emphasized that, the time spent on fetching water was between 10 to about 40 minutes. It was observed that the unprotected springs which also served as water source to many were located next to the river since they also fed into the same river. Therefore these water springs were about the same distance from the camp as the river.

**Figure 2:**
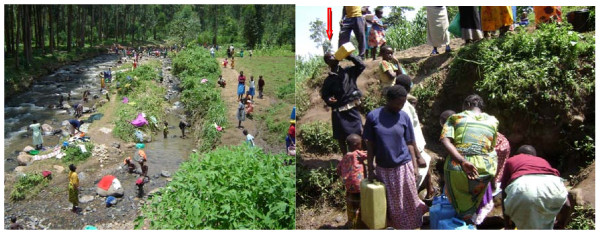
**Photographs of Human activity and water use in River Manafwa**.

The question of perceptions about water safety and reliability was discussed. Majority of the KIs and FGD participants were of the opinion that tank water was most safe. Inspiteof this positive perception, this water was reported to have a smell. Discussions suggested that this was probably due to the water purifier known as 'water guard'. According to KIs, river Manafwa water was considered by local residents and camp inhabitants to have better taste. Findings from observations concurred with those of informants that river water appeared dirty with a lot of activities around, suggesting that the water was likely to be contaminated (Figure 2).

"River Manafwa is highly contaminated but it is being used by people especially children. Children swim in the water and they drink that same water." (District Health Official)

The most reliable source was the river according, to most FGDs and KIs. Tank water was mentioned to be unreliable, by KIs mostly from health department and Local Government councils. Observations also showed that sometimes, these water tanks were empty, leaving people with no other alternative water sources than the river and unprotected water springs. However, KIs reported that tank water presumed to be safe water would not be enough for the population in the long term.

"We are afraid that water may not be available to people in the long run because the main source of water is from Mbale. I am sure the Government will not truck water for a long period of time." (District Health official)

"This water is not enough either for short or long term needs of the affected population. Water from the tanker will be available for a very short time. So there is need to repair the gravity water stand pipes so that people can have water for a longer period of time" (Local Council official)

Qualitative data was collected on household water collection, treatment and storage.During observations, most people were seen using 20 litre plastic containers both for water collection and storage. In a survey of 397 households, results showed that most (91.7%, 364/375) of households stored drinking water in 20 litre plastic containers. A small number of people stored their drinking water in buckets (7.8%, 31/375), pots (0.8%, 3/375) and saucepans (0.3%, 1/375) respectively. On the other hand 5.5% (22/397) of household heads indicated that they did not store drinking water at all.

Data from FGD showed that water was being treated at tent level with purifiers such as water guard. This responsibility was given to the tent leaders to treat drinking water for all the households in the respective tents. Findings from the quantitative survey indicated that 74.3% of households had their water treated while 73.6% had covered drinking water storage containers.

Our study established that there were a number of traditional beliefs that govern water use and storage. A number of water related beliefs were mentioned by key informants. One of the beliefs is that un-boiled river water is very tasty.

"These people believe that river water is very tasty. As a result of this, some people are still drinking river water." (KI, Health official).

This community has people who do not like the taste of treated water. Our people also say unboiled water tastes better than boiled water so most of them do not boil water. About 8/10 prefer unboiled water to boiled." (KI, Local Council official)

Besides, storage of water in pots is a valued tradition. Key informant data also indicates that women should be the ones to fetch water. This perception keeps most household burden on women.

"According to our tradition, women are supposed to collect water from the well. We also used to store our drinking water in the water pots but now we are forced to keep our drinking water in the Jerrycans. We are not comfortable with this practice but we have no way out." (KI Local Council official)

In order to provide safe water, several interventions were instituted. The notable interventions in place to provide safe water included; supply of safe water from NWSC from Mbale, provision of Water Guard for water treatment, provision of water collection and storage containers (like 20-liter plastic containers and buckets). There was ongoing health education by health workers from the district health department and relief agencies. Fencing off the water sources (tanks and stand-pipes) had also been done. Good progress was also reported to have been made on repairing the gravity water scheme to provide a reliable longer term source of water supply. Observations also confirmed that the gravity flow water scheme was being worked on.

### Sanitation and hygiene needs in the camp

The main excreta disposal facilities available at the camp were pit latrines. By the time of this assessment (approximately two weeks after the landslide) there were 23 operational pit latrine stances in place. According to KI interviews, the water table in the area is high, which affected the sinking levels of the pits. As a result the pit latrines were shallow and this contributed to the bad smell of latrines. Some KIs estimated the depth of latrines to be about 8 to 10 feet.

"These latrines are few and shallow. The water table is very high, maybe this is the reason why these latrines smell badly after only one week of use."(FGD Male)

The pit latrines' floors were made of timber and Sanplasts. The super- structure was made of timber and covered with tarpaulins. Latrines were difficult to clean mainly because of non-cemented floors. Observations showed that the use of timber floors made it difficult to keep them clean. The latrines were not segregated for sexes and had limited privacy. The entrances were covered with tarpaulins flaps. Inadequate latrine facilities seemed to be a major problem affecting people living in the camp. This was evidenced by the responses from most of the key informants who complained that there were only 23 latrine stances for over a large number of people. A similar observation was made by the focus group participants who also noted that pit latrines were not enough and had limited privacy. Focus groups showed that adults were not comfortable with turplins that were used as doors and walls.

"These latrines have no doors except the curtains. We actually noticed that the adults were not using the latrines during day time due to lack of privacy."(KI District health worker)

This was re-echoed in the FGDs that:

"These latrines are not enough because we are so many people and during morning hours we are so crowded and almost line up to use them". (FGD Male)

Status of the pit latrines: It was noted by three of the four FGDs and majority of the KIs that latrines were littered with feces, smelling badly and not clean. The survey indicated flies (70.0%, 278/397), littering of feces in and around the latrines (59.7%, 237/397) and smell (81.4%, 323/397) as the major nuisances in these latrines. Also noted was absence of lighting in the latrines or latrine areas at night. This made it difficult for people to use latrines especially at night. This factor reportedly resulted into people defecating in the surrounding bushes or on the slabs as indicated in the quote below:

"Among the heaps of feces we find in the morning, there are those that are big indication that they are from adults not children. I think this problem of lights also affects us adults. It also seems like among us there are people who have not been using latrines in the places where they have been and have continued with the same practice of openly defecating any where they feel like." (FGD Male)

"Here they use latrines all the time during the day but at night it is very difficult to use because there is no light in these latrines". **(**KI Health worker)

Furthermore, data from key informants showed that the latrines were on the upper side of the water sources (river and unprotected water springs). They, however, noted that there was no risk of water contamination because the water sources were at a distance. Observation, however, showed that there was a risk of contamination given that the pit latrines are very shallow and the topography was quite steep. Since it was constantly raining, the feces deposited in the bushes could be washed into the river and protected spring.

### Sanitation and hygiene practices in the camp

Qualitative results from most KIs and FGDs show inconsistent use of latrines. It was reported that children below five years did not independently use latrines or they 'visited' the nearby bushes. They were either escorted by elder siblings or mothers to avoid misuse of the latrines. The toddlers on the other hand defecated on bare ground or on leaves or pieces of paper put on the ground and the adults, usually the mothers, disposed of the excreta in the latrines. Observation data confirmed these findings. Survey results showed that (94.5%) people threw children's feces in a pit latrine. The table below shows details on other practices regarding handling of children's feces (Table 2).

**Table 2:** Practices regarding handling of children's feces

Variable	Frequency (n)	Percent (%)
Defecation facilities for children		

On the ground in the tent	77	19.8

On the ground outside the tent	178	45.8

In the latrine	184	47.3

Disposal of children's feaces		

In the latrine	353	94.5

Left on the ground	6	1.6

Thrown outside	3	0.8

Buried in the ground	8	2.1

Children's anal cleansing after defecation		

Yes	354	91.0

No	35	9.0

Materials used for children's anal cleansing		

Leaves	198	55.9

Ordinary paper	99	28.0

Water	107	30.2

Others (toilet paper, sliding and rags)	70	19.8

"We get something like a piece paper, a leaf or polythene which we lay on the ground for the children to defecate then we take and throw in the latrines. In case a child defecates on the ground, we get a hoe and dig out that part then we throw in the latrine". (FGD females)

"For the children who are below five years, their mothers put for them something on the ground to defecate on and they take to the latrine. However, we have a problem getting papers or leaves or anything for the mothers to put on the ground for the children to defecate on before taking the fecal discharge to the latrine."(FGD Male)

A number of beliefs and practices related to latrine use were revealed by the FGDs and KIs. It was reported that men and women in this community do not share latrines, in-laws do not share latrines, different family members do not share latrines, and that children's excreta is harmless. Some of the vivid quotes demonstrate this.

"Women and men do not share latrines. In laws also do not share latrines. They also think children's feces are not harmful and as a result of this, children are left to defecate anywhere. They can even use their bare hands to clean children's feces." (KI District health worker)

Such practices pose possible threat for disease outbreak. Respondents felt that their privacy rights were being violated by sharing latrines with very many other families.

We assessed a number of hygiene practices in the camp. These were; hand washing with soap after latrine use, storing/covering drinking water in clean containers, covering food, cleaning of latrines and anal cleansing.

It was noted by KIs that hand washing with soap after latrine use was very new to most people. Very few people therefore washed their hands after defecation. Most of the camp leaders mentioned that soap and water were available at latrines. However, 3 of the 4 focus group revealed that hand washing facilities were not adequate. In household interviews, results show that 90.2% (358/397) of respondents indicated that they always washed their hands after visiting a latrine and 7.3% (29/397) said they sometimes washed their hands while 2.5% (10/397) said they did not wash their hands after visiting a latrine. Of those who said they always and sometimes washed their hands after visiting a latrine, 38.5% (149/387) said they used water only, 76.5% (296/387) said they used water with soap. After handling of children's feces, 81.1% (287/354) of respondents interviewed said they washed their hands with water and soap while 26.3% (93/354) of people said the washed their hands with water only. It was also observed that a number of women did not bother to wash their hands after disposing/handling children's feces. When stratified by sex, it was observed that males were three times more likely to say that they washed their hands after visiting a latrine with water and soap (OR 3.584, 95% CI 1.658 - 7.748). On the other hand after handling or disposing of children's fences hand washing with water and soap was again significantly associated with being a male (OR 3.157, 95% CI 1.307 - 7.623) (Table 3).

**Table 3:** Water, sanitation and hygiene practices by sex

Variable	Males (%)	Females (%)	OR	95% CI
Drinking water stored in covered container				

Yes	71(83.5)	221(76.2)	1.583	0.840 - 2.984

No	14(16.5)	69(23.8)	1.0	

Drinking water treated				

Yes	73(85.9)	247(85.2)	1.059	0.531 - 2.114

No	12(14.1)	43(14.80	1.0	

Household members use latrine				

Yes	82(96.5)	298(95.5)	1.28	0.35 - 7.13

No	3(3.5)	14(4.5)	1.0	

Wash hands with Water and Soap after Visiting a latrine				

Yes	76(25.7)	8(8.8)	3.584	1.658 - 7.748*

No	220(74.3)	83(91.2)	1.0	

Where children Defecated				

In the pit latrine	40(50.6)	185(64.2)	0.571	0.345 - 0.944*

Other areas	39(49.6)	103(35.8)	1.0	

Wash hands with soap and water after handling child's feaces				

Yes	68(91.9)	219(78.2)	3.157	1.307 - 7.623*

No	6(8.1)	61(21.8)	1.0	

*= Statistically significant findings

Observations also revealed that water and soap were inconsistently available for use after visiting a latrine. This was more common in the afternoons than in the mornings. In fact expressions of frustration were visible on finding no water to wash hands.

Materials for anal cleansing after latrine use were mostly plant leaves according to FGD and KIs. Some people mentioned toilet paper as another material used for anal cleansing. Responses from KIs and majority of the FGD participants showed that some people did not have anal cleansing materials.

"Some people do not bother. They just walk in and out of the latrines without cleansing." (FGD Female)

"We do not have any anal cleansing material in the latrines and we are doing very badly in this aspect. However, we improvise with certain leaves which are also not easy to find". (FGD Males)

Observations were made on bathrooms, drainage situation, cooking facilities and solid waste management. Sanitation observations revealed that some bathrooms (about 10) were erected specifically for women. But these lacked soak pits. These bathrooms had just been opened and therefore a problem of poor waste water drainage was anticipated. Clearly these were inadequate. No bathrooms were in place for males at the time of the study. Besides, Key informants reported that there was a big problem with drainage|. After a heavy rain, it was reported that the whole camp gets flooded even in the tents Key informants further revealed that whenever it rained heavily water flooded causing a big problem since tent floors get wet. In some cases, runoff water from the tent roofs was very discomforting as it flooded and settled in some tents. Particularly, the children under five years would have nowhere to rest completely. No kitchens were operational at the time of assessment. However, some were being erected. Food was being prepared in the open and under trees. This caused a lot of difficulties in food preparation especially when it rained. Hygiene maintenance during food preparation was therefore rendered difficult.

On solid waste management, the major types of solid waste observed in the camp were leftover food, peelings, polyethylene and plastics such as bottled water containers. Three rubbish pits had been provided by the Uganda Red Cross Society for disposal of solid wastes. However the proximity of these open pits to the food preparation areas was likely to attract flies and bad smell. However it was reported that the pits were left open for short periods, once filled they are covered and new ones opened.

In terms of hygiene and sanitation interventions, there were a number of hygiene and sanitation interventions being carried out in the camp. Interventions mentioned by FGD participants and key informants included; Digging of the pit latrines by partners (OXFAM, Uganda Red Cross Society-URCS), health education by District health educators and CORPS, supply of safe water by Government and partners, digging rubbish pits by URCS, health inspection of the camps, voluntary cleaning of the latrines by camp residents and provision of hand washing facilities by NGOs.

### Vector control and vector borne diseases

Vector borne diseases especially malaria and diarrheal diseases can become a major problem among displaced populations. We therefore investigated the risks of acquiring vector borne diseases in these camps. Mosquitoes and flies were the major vectors reported, although some key informants indicated that there was no evidence of vector breeding sites at the camp area. Overall, 48% (135/283) had experienced at least one member of the household falling sick since moving to the camp, and most KIs attributed the illness to Malaria fever. (See table 4)

**Table 4:** Main causes of morbidity in the camp 2 weeks after the disaster

Variable	Frequency (n = 283)	Percent (%)
Malaria	135	47.7

Diarrhea	25	8.8

Stomach ache	18	6.4

Respiratory infections	165	58.3

Our study established that community perspectives on the relationships between vectors and diseases. According to KIs, some people are not able to link the vectors with diseases causation. The following are quotations demonstrate this view.

"People don't know that mosquitoes transmit malaria. Some still think that malaria is caused by rain". (Local Council official)

"These people were not protecting themselves from mosquito bites. Some people believe that nets are for dead people." (KI Local Council official)

A number of interventions to control vectors and vector borne diseases were put in place to control mosquitoes. Children under five years and pregnant women were provided with Insecticide Treated Nets (ITNS) by the Uganda Red Cross Society. However, there was no evidence of nets hanged in the tents by the time of this study. Sensitization on the use of the ITNS was still low as most of the respondents had not received messages on ITNS. Some of nets were observed still in their packets.

"Some people were freely given mosquito nets while others were not. But even those who were given do not use them because they do not have anywhere to hang them. The people who gave the nets targeted pregnant mothers and children who are less than five years old. These nets are already treated. We were told to first spread them for air to pass through before use but most of us haven't used them and they are still packed as they were given to us." (FGD males)

As part of the vector control, the environment health workers deployed at the camp were encouraging the burning of garbage, digging of rubbish pits, covering of foodstuff, cleaning of the drainage channels for water and slashing the nearby bushes around the tents. The community health workers under the Foundation for Development of Needy Communities were actively participating in educating the communities on the basics of sanitation and hygiene. Some of the control activities were, however, hampered by the limited supply of tools for use like spades, protective wears, hoes and rakes for cleaning.

"We would be doing a good job of cleaning with our people but we lack protective wear, hoes and other items" (KI Development Partner)

"On the side on cleaning, we need to be provided with some gloves and other protective wear so that we do not contract disease. Sometimes the health worker who come to oversee the cleaning come when they have put on gloves but us who do the cleaning do not put on anything while we are cleaning." **(**FDG females)

## Discussion

While disaster response measures have been standardized by international agencies that provide excellent services to displaced populations, the actual effectiveness of interventions may be influenced by environmental, behavioural and cultural practices of the affected population. This assessment, conducted two weeks after the camp was set up, provides insight into the situation of water, sanitation and hygiene, and vector control in Bulucheke camp. The assessment indicates strong traditional beliefs governing water use, and human excreta disposal, which could potentially compromise the effectiveness of the interventions. There were inadequate hygiene facilities. This situation influenced people's sanitation and hygiene behaviours.

Outbreaks of diarrheal diseases, particularly dysentery and cholera, are a great concern where a population is living in a crowded environment such as a camp [9-11], hence provision of safe water supply is one of the rapid response actions by relief agencies. Although there was evidence that efforts have been to this effect, our assessment established that safe water was not readily accessible as it was irregular. It was evident that the camp population was utilizing River Manafwa and unprotected spring water for domestic purposes i.e. cooking and drinking. The same river was being used for laundry, bathing and recreation (swimming) especially for the children (Picture 1). Combined with the traditional beliefs that 'un-boiled water tastes better", this could lead to a potentially dangerous situation that could easily lead to outbreaks of diarrheal diseases [12].

The population per latrine ratio in the camp was 217 people per latrine stance. This is far below the Minimum Sphere Standard which requires a maximum of 50 people per latrine in areas where there are no latrines initially (like in this case) and decreasing to 20 people per latrine stance as soon as possible [13]. Moreover, we found traditional beliefs that prohibit latrine sharing among certain groups of people such as in-laws or other families. These beliefs are not only unique to Bududa, but similar taboos, cultural and customary beliefs deeply affect sanitation in most parts of rural Uganda [14]. Such beliefs need to be corrected through intensive mobilization and education at grassroot level.

The latrines were also very dirty, littered with feces, smelly, and infested with flies. These factors obviously impedes use and de-motivates latrine users [15]. Absence of lighting in the latrines further compromised their use. Light is essential for accurate aiming of feces inside the pit always fear of stepping onto feces, and reduces fear of unknown dangers in the darkness surrounding the toilet. Children and adolescents that would fear to visit the latrine due to darkness get motivated. Besides, females who otherwise would fear sexual violence such as rape as they visit latrines at night get protected.

Control of vectors is essential as it affects health and wellbeing of the people. The high percentage of households reporting malaria cases and other fevers was probably due to poor utilization of preventive measures availed to the camp occupants as well as the camp living situation itself. One can argue that the incidence of malaria could even be higher since the incubation period mostly ranges from 7 days to 4 weeks [16]. Although mosquito nets had been provided to children under 5 and pregnant mothers, they were hardly used, largely due to lack of facilities to hang the nets on. There is therefore need for innovations that enable using nets in tents to avert morbidity due to malaria. More health education on vector control with emphasis on increasing knowledge to counteract beliefs such as 'nets are for dead people" is critical

## Conclusions

A number of agencies such as WHO, UNICEF, Ministry of Health, URCS and Oxfam helped tremendously to mitigate the situation. There was inadequate access to safe water in the camp. Most people were therefore exposed to water related diseases since they used river and unprotected spring water which was potentially unsafe. The belief that safe water had bad taste and unsafe water was tasty needed a health education intervention. In terms of sanitation and hygiene, pit-latrines were inadequate, poorly maintained and not user-friendly for most people. There was a gap between health education and actual practice for some people regarding sanitation and hygiene practices. Traditional beliefs and practices that hinder latrine use needed correction. There were attempts to control mosquitoes through provision of ITNs but most people were not using them due to traditional beliefs and mechanisms of hanging them in the tents. Therefore, the most vulnerable people were at risk of acquiring malaria disease.

We, therefore, recommend that responsible authorities should design a means of increasing and sustaining access to safe water. Besides, strengthening community health education on the dangers of using unsafe water sources like the river and unprotected spring is critical, and traditional beliefs and myths need to be addressed. Focus should be on bridging the gap between what people know about water, sanitation and hygiene and their actual practices. Construction of more pit latrines to match the high population in the camp with improved structure and privacy would improve sanitation situation. The pit latrines should be segregated for women and men with adequate lighting at night. Camp leaders, health workers and other partners should ensure that hand washing facilities are continually operational by making sure that water and soap are available. Provision of liquid soap in lieu of bar soap at hand washing points would also improve hygiene situation. Measures should be put in place to involve the community (affected population) in maintenance of water sources, hand washing facilities and pit latrines.

## List of abbreviations

DHO: District Health Officer; FGDs: Focus Group Discussions; IDP: Internally Displaced; KI: Key Informant Persons; LIPHEA: Leadership Initiative for Public Health in East Africa; MakSPH: Makerere University School of Public Health; MoH: Ministry of Health; NGO: Non-governmental organizations; NWSC: National Water and Sewerage Corporation; RAs: Research Assistants; UNICEF: United Nations Children's.

## Competing interests

The authors declare that they have no competing interests.

## Authors' contributions

LMA and ME contributed to the study concept and design, data collection and supervision, analysis and write up of the manuscript. CGO, MM and WB contributed to the study conceptualization and design, and manuscript writing. All authors read and approved the final manuscript.

## Appendix 1: An example of some of the questions asked in the Focus Group Discussion and Key Informant Guide

An example of some questions in the Focus Group Discussion guide

1. Where do you collect your water from for drinking, washing, cooking? Who collects the water?

2. What do you think of the water that you use? Probe for taste, quality, distance, color

3. How long do you think one spends to collect water?

4. Where do most families in this camp store drinking water?

5. Where do most families wash your clothes from?

6. Would you say you have enough water? Explain

Latrine Use

1. In the new settlement (camp), do you have latrines? If yes, what type of latrines?

2. How do you find the latrines? (structures, cleanliness, privacy, smell etc.)

3. Are there times when people do not use a latrine? If yes, explain

4. Would you say that young children use the latrines? From what age do children start to use the latrine?

5. How is stool of children disposed?

6. Who provided the latrines? Who cleans, repairs, empties the latrines?

7. Do young children wash their hands after using the latrine?

8. Do adults wash their hands after using the latrine?

9. Suggest ways on how latrine use can be improved?

An example of some questions in the Key informant guide for Bududa district leaders*

1. What are the current main health problems in this camp and the communities?

2. Are you aware of any resettlement measures? If yes, describe them.

3. How many people do you estimate to be in the camps currently?

4. With regard to the people in the camps, do you know where they are likely to migrate to? Are there any challenges envisaged? Explain

5. What are the current or threatened water and sanitation-related diseases?

6. Who are the most vulnerable people in the population and why (explain)?

7. Is there equal access for all to existing facilities such as pit latrines, birth rooms etc? Explain

8. What special security risks exist for women and girls?

9. What water and sanitation practices were the population accustomed to before the emergency? What about now in the camp?

10. What do you think are the priority health problems if one is to intervene?

In your opinion, what strategies would you recommends to mitigate future disasters in this district

*We had different types of key informants and they had specific suitable tools
